# Assessment of estimated glomerular filtration rate based on cystatin C in diabetic nephropathy

**DOI:** 10.1590/2175-8239-JBN-2020-0145

**Published:** 2021-02-12

**Authors:** Kadriye Akpınar, Diler Aslan, Semin Melahat Fenkçi

**Affiliations:** 1 Pamukkale University, Faculty of Medicine, Department of Medical Biochemistry, Denizli, Turkey.; 2 Pamukkale University, Faculty of Medicine, Division of Endocrinology and Metabolism, Department of Internal Medicine, Denizli, Turkey.

**Keywords:** Diabetes Mellitus, Type 2, Diabetic Nephropathies, Glomerular Filtration Rate, Cystatin C, Diabetes Mellitus Tipo 2, Nefropatias Diabéticas, Taxa de Filtração Glomerular, Cistatina C

## Abstract

**Introduction:**

GFR is estimated by using creatinine and cystatin C to determine renal dysfunction. Our aim was to evaluate estimated GFR (eGFR) based on cystatin C in type 2 diabetic patients with diabetic nephropathy (DN).

**Methods:**

Study group included 52 controls (46% male, age: 54.5±12.4) and 101 diabetic patients (46.5% male, age: 58.2±11). The diabetics were divided into three subgroups according to 24-hour urine albumin: normal to mildly increased (A1) (n=51), moderately increased (A2) (n=25), severely increased (A3) (n=25) albuminuria. Creatinine clearance (CrCl) was determined. Correlations between CrCl and eGFRs estimated according to the CKD-EPI, MDRD, and Cockcroft-Gault (CG) formulas, and ROC curves were evaluated. Data were analyzed using SPSS 22.0.

**Results:**

Only CKD-EPI-cys eGFR was significantly lower in the A1 group than the controls (p=0.021). All GFRs were lower in the A3 group than the control (CKD-EPI-cr, MDRD, CKD-EPI-cys, CKD-EPI-cr-cys: p=0.0001, CG and CrCl: p=0.001) and A1 (for all GFRs p=0.0001) groups. CKD-EPI-cr (p=0.004), MDRD (p=0.01), CG (p=0.037), CKD-EPI-cys (p=0.033), and CKD-EPI-cr-cys (p=0.016) eGFRs in the A2 group were significantly different from the A1 group. All eGFRs showed a moderate correlation with CrCl in the A1group (CKD-EPI-cr and CKD-EPI-cr-cys: r=0.49, p=0.0001, MDRD: r=0.44, p=0.001, CG r=0.48, p=0.0001: CKD-EPI-cys r=0.40, p=0.004). The area under the CKD-EPI-cys ROC curve was the highest and found to be 0.847 (95%CI 0.763-0.931, p=0.0001).

**Conclusions:**

Our results showed that the CKD-EPI-cys eGFR can be useful in detecting the early stage of DN and more predictive than the others for prediction of DN.

## Resumo

**Introdução:**

A TFG é estimada usando creatinina e cistatina C para determinar a disfunção renal. Nosso objetivo foi avaliar a TFG estimada (TFGe) com base na cistatina C em pacientes com diabetes do tipo 2 com nefropatia diabética (ND).

**Métodos:**

O grupo de estudo incluiu 52 controles (46% homens, idade: 54,5±12,4) e 101 pacientes diabéticos (46,5% homens, idade: 58,2±11). Os diabéticos foram divididos em três subgrupos de acordo com a albumina na urina de 24 horas: albuminúria normal a levemente aumentada (A1) (n=51), moderadamente aumentada (A2) (n=25) e severamente aumentada (A3) (n=25). Foi determinado o clearance de creatinina (Clcr). As correlações entre Clcr e TFGe calculadas de acordo com as fórmulas CKD-EPI, MDRD, e Cockcroft-Gault (CG), e as curvas ROC foram avaliadas. Os dados foram analisados usando o SPSS 22.0.

**Resultados:**

Somente a TFGe CKD-EPI-cis foi significativamente menor no grupo A1 do que nos controles (p=0,021). Todas as TFGs foram mais baixas no grupo A3 do que no grupo controle (CKD-EPI-cr, MDRD, CKD-EPI-cis, CKD-EPI-cr-cis: p=0,0001, CG e Clcr: p=0,001) e no grupo A1 (para todas as TFGs p=0,0001). As TFGes CKD-EPI-cr (p=0,004), MDRD (p=0,01), CG (p=0,037), CKD-EPI-cis (p=0,033), e CKD-EPI-cr-cis (p=0,016) no grupo A2 foram significativamente diferentes do grupo A1. Todas as TFGes mostraram uma correlação moderada com Clcr no grupo A1 (CKD-EPI-cr e CKD-EPI-cr-cis: r=0,49, p=0,0001, MDRD: r=0,44, p=0,001, CG r=0,48, p=0,0001: CKD-EPI-cis r=0,40, p=0,004). A área sob a curva ROC CKD-EPI-cis foi a mais alta e foi considerada 0,847 (95%IC 0,763-0,931, p=0,0001).

**Conclusões:**

Nossos resultados mostraram que a TFGe CKD-EPI-cis pode ser útil na detecção do estágio inicial de ND e com maior valor de predição do que as outras para a predição da ND.

## Introduction

Glomerular filtration rate (GFR) is the flow rate in milliliters per minute of the plasma that substances are freely filtered from kidney glomeruli membranes^1^. GFR is considered the best indicator for kidney function. The gold standard method for assessing GFR is the renal inulin clearance. However, as an exogenous substance, inulin is not suitable for daily practice^2^. Creatinine and cystatin C are endogenous markers used in the estimation of GFR^3^. Creatinine is a convenient and inexpensive marker for GFR but is affected by age, gender, exercise, muscle mass, and diet^4^. One of the most widely used assessment methods for GFR is the 24-hour creatinine clearance (CrCl). However, because it is time-consuming and the collection of 24-hour urine is not precise, some useful formulas have been produced for estimation of GFR (eGFR) by means of the serum creatinine or/and cystatin C levels. These formulas are shown in Chart 1

**Chart 1:** Creatinine and cystatin C-based equations for GFRs^5^^,^ 6 ^,^ 7

**CrCl (mL/min/1.73 m^2^)** = [Ucr/Scr] × [24 hour urine volume (mL)/1440] × [1.73/BSA]
Ucr is urine creatinine (mg/dL), Scr is serum creatinine (mg/dL). BSA (body surface area) is calculated using DuBois formula: BSA = (W ^0.425^ x H ^0.725^) x 0.007184
**MDRD-eGFR (mL/min/1.73 m^2^)** = 175 × (Scr) ^-1.154^ × (Age) ^-0.203^ (× 0.742 if female) (× 1,212 if black)
Scr is serum creatinine (mg/dL).
**2009 CKD-EPI- cr eGFR (mL/min/1.73 m^2^)** = 141 × min (Scr/κ,1)^α^ × max (Scr/κ, 1)^-1.209^ × 0.993^Age^ × 1.018 [if female] * 1.159 [if black]
Scr is serum creatinine (mg/dL), *κ* is 0.7 for females and 0.9 for males, *α* is -0.329 for females and -0.411 for males, min indicates the minimum of Scr/*κ* or 1, and max indicates the maximum of Scr/*κ* or 1.
**2012 CKD-EPI cys C eGFR (mL/min/1.73 m^2^)** = 133 × min(Scys/0.8, 1)^-0.499^ × max (Scys/0.8, 1)^-1.328^ × 0.996^Age^ × 0.932 [if female]
Scys is serum cystatin C (mg/dL), min indicates the minimum of Scys/0.8 or 1, and max indicates the maximum of Scys/0.8 or 1.
**2012 CKD-EPI cr-cys C eGFR (mL/min/1.73 m^2^)** = 135 × min(SCr/ *κ*, 1)^α^ × max(SCr/κ, 1)-0.601 × min(Scys/0.8, 1)^-0.375^ × max(Scys/0.8, 1)^-0.71^× 0.995^Age^ ×0.969 [if female] ×1.08 [if black]
Scr is serum creatinine (mg/dL), *κ* is 0.7 for females and 0.9 for males, *α* is -0.248 for females and -0.207 for males, min(SCr/ *κ*, 1) indicates the minimum of Scr/*κ* or 1, and max(SCr/κ, 1) indicates the maximum of Scr/κ or 1, min(Scys/0.8, 1) indicates the minimum of Scys/0.8 or 1, and max(Scys/0.8, 1) indicates the maximum of Scys/0.8 or 1.
**Cockcroft-Gault (mL/min/1.73 m^2^) eGFR** = [140-Age × Body weight (kg)] × 0.85 (if female) / Scr × 72
Scr is serum creatinine (mg/dL).

* To convert Scr values in µmol/L to mg/dL, divide by 88.4.

5 ^,^ 6 ^,^ 7.

Diabetic nephropathy (DN) is a pathological clinical syndrome characterized by urinary albumin excretion in diabetic patients, associated with glomerular lesions and loss of GFR. The incidence of DN increases over time and leads to chronic kidney disease (CKD) (12-55%) 8 ^,^ 9.

Patients with CKD have persistent albuminuria (>300 mg/24-hour or >20 µg/dk), and usually their eGFRs are below <60 mL/min/1.73m^2^. Urine albumin levels and eGFRs should be evaluated at least once a year in patients with type 2 diabetes with comorbid hypertension and on those with type 1 diabetes for more than 5 years^6^. According to the American Diabetes Association (ADA), creatinine-based eGFR estimated by the Modification of Diet in Renal Disease (MDRD) or Chronic Kidney Disease Epidemiology Collaboration (CKD-EPI) formulas can be used for the evaluation of GFR in patients with DN^10^.

Cystatin C is a low molecular weight protein that is an endogenous cysteine proteinase inhibitor and has a high correlation with GFR. This correlation is independent of inflammatory conditions, muscle mass, gender, body composition, and age (after 12 months). Unlike creatinine, it does not have a tubular secretion. Serum and urine cystatin C levels are higher in type 2 DN. There are several studies showing that cystatin C performs better than creatinine as an indicator of GFR in chronic kidney disease, and it is superior to other markers, especially in patients with eGFR <60 mL/min/1.73m^2^, diabetic children, changes in muscle mass, liver diseases, and the elders^11^^,^ 12 ^,^ 13.

In this study, we aimed to evaluate CKD-EPI-cys eGFR in patients with type 2 DN by comparing with creatinine clearance, CKD-EPI-cr, MDRD, CG, and CKD-EPI-cr-cys eGFRs formulas.

## Materials and Methods

### Subjects

Fifty two healthy controls aged ≥18 years [n= 52, age: 54.5 (SD: 12.4)] and 101 type 2 diabetic patients admitted to the Endocrinology and Metabolism outpatient clinic in Medical Faculty of Pamukkale University, between December 2017 and May 2018 [n= 101, age: 58.2 (SD: 11)] were included in our study. Exclusion criteria comprised chronic use of corticosteroids, significant obesity (BMI>35 kg/m^2^), pregnancy, renal diseases other than DN, malignancy, infection, and thyroid disorders for all subjects and medication use for healthy volunteers.

Height, weight, body mass index (BMI= weight (kg)/height (m)^2^), systolic blood pressure (SBP), diastolic blood pressure (DBP), medical history including duration of diabetes, smoking and alcohol use of patients and controls were recorded. Body surface area (BSA) was calculated using the DuBois formula^14^. The diabetics were divided into three subgroups according to 24-hour urine albumin: normal to mildly increased (A1) (n= 51); moderately increased (A2) (n= 25); and severely increased (A3) (n= 25) albuminuria. The diagnosis of DN was made by the clinician according to GFR and albuminuria categories, other risk factors, and comorbid conditions^6^. All procedures involving participants and data were in accordance with the revised Helsinki Declaration of 2000 and the study was approved by Pamukkale University Medical Ethics Committee (No. 13, Date: 03.10.2017).

### Methods

Venous blood samples were taken from patients in sitting position in the morning, after 8-12 hours of fasting, into gel vacuum tubes for biochemistry (Vacusera, Turkey), and into whole blood tube with EDTA (ethylenediaminetetraacetic acid) (Vacusera, Turkey) for HbA1c and hematocrit anaylsis. Twenty-four-hour urine specimens were collected from the participants after the essential instructions. The measurements were performed at the Biochemistry Laboratory in Medical Faculty, Research and Application Hospital in Pamukkale University. Total protein (sTP), albumin (sAlb), creatinine (sCr), and cystatin C (sCys C) in serum, and HbA1c and hematocrit (Hct) in whole blood, and protein (uTP), albumin (uAlb), and creatinine (Ucr) in urine were measured.

Serum urea and creatinine levels were measured by the kinetic colorimetric method (the "compensated" Jaffé assay for creatinine has been standardized against the isotope dilution mass spectrometry (IDMS) traceable values) and serum cystatin C was measured by particle enhanced immunturbidimetric assay (PETIA) on autoanalyzer (Cobas 8000, Roche Diagnostics GmbH, Mannheim, Germany). Urine protein and albumin were analyzed by immunoturbidimetric assay, and urine creatinine was analyzed by kinetic colorimetric method on autoanalyzer (Cobas 8000, Roche Diagnostics GmbH, Mannheim, Germany). HbA1c was studied by HPLC, ion exchange method (Tosoh G8 Bioscience, USA). Hematocrit was measured by hematology analyzer (Mindray BC 6800, China). For internal quality control, two levels of assayed quality control materials were tested once a day. Two levels of internal quality controls provided by kit manufacturers (Bio-Rad, Hercules, CA, USA) were routinely analyzed once a day, and the external quality control program material (Bio-Rad, Hercules, CA, USA) were analyzed monthly. All of the results were acceptable during the study.

The GFRs were estimated using creatinine clearance, CKD-EPI based on creatinine or/and cystatin C, MDRD and Cockcroft-Gault (CG) formulas seen in Chart 1 5 ^,^ 6 ^,^ 7.

### Statistical Analysis

The study population was determined using G*Power 3.1 (Foul, Erdfelder, Lang and Bucher, 2007) program. According to the reference study results^15^, the variables had a large effect size (F=0.725). Assuming we can achieve a lower effect size level (F=0.5), a power analysis was performed before the study. Accordingly, including at least 76 subjects (19 for each group) in the study would result in 95% power with 95% confidence level. Considering the possible loss of subjects, 30% more subjects were included in each group and the study was completed with 25 people in DN subgroups.

Patient information (age, gender, race, height, weight, blood pressure, medical history) and the biochemical/hematological test results were evaluated after all diabetic patients were divided into three subgroups according to 24-hour urine albumin levels: normal to mildly increased (A1) (<30 mg/24 h), moderately increased (A2) (30-300 mg/24 h), and severely increased (A3) (>300mg/24 h) albuminuria, and the results were compared between these subgroups and healthy individuals. Continuous variables were expressed as mean ± standard deviation (SD) or medians and quartiles, and categorical variables as frequencies and percentages. The data were tested for deviation from Gaussian distribution using the Kolmogorov-Smirnov test. When parametric test assumptions were met, one-way anova test was used for comparison of independent group differences. Otherwise, Kruskal Wallis test was used to compare independent group differences. The differences between groups were considered significant if p value was less than 0.05 (two-tailed). Correlations between CrCl and eGFRs were evaluated according to Spearman r correlation coefficient (r value: 0.00-0.49 low, 0.50-0.69 moderate, ≥0.70 high). GFRs were compared using Receiver Operating Characteristic (ROC) curve analysis. All data were analyzed using the SPSS 22.0 program (SPSS, Chicago, USA).

## Results

The mean age of the controls (n= 52) was 54.5 ± 12.4 and the mean age of diabetic patients (n= 101) was 58.2 ± 11. Forty-six percent of the controls and 46.5% of the diabetics were male. There was no difference between patients and control groups in age and gender.

The patients were subdivided according to their albuminuria status. Characteristics of study participants, and biochemical measurement results are shown in Table 1.

**Table 1:** Clinical and biochemical characteristics of controls and patients with type 2 diabetes with normal to mildly increased (A1), moderately increased (A2), and severely increased (A3) albuminuria

	Control (n=52)	A1 (n=51)	A2 (n=25)	A3 (n=25)	P value
**Age (years)**	54 (12)	57 (10)	61 (12)	56 (11)	0.114
**Male (n, %)**	24, 46	18, 35	12, 48	17, 68	0.064
**Race-White (%)**	100	100	100	100	
**Body Weight (kg)**	75 (64-84)	80 (72-89)	82 (70-92)	82 (70-98)	0.051
**BMI (kg/m^2^)**	25 (23-27)	31 (28-34)	31 (28-35)	29 (26-33)	0.0001
BSA (m^2^)**	1.85 (0.18)	1.83 (0.17)	1.88 (0.19)	1.92 (0.20)	0.25
**SBP (mmHg)**	120 (110-120)	125 (120-140)	130 (120-142)	130 (120-150)	0.0001
**DBP (mmHg)**	80 (70-80)	75 (65-80)	80 (70-80)	75 (70-80)	0.621
**Current Smoker (n, %)**	0, 0	5, 9	4, 16	3, 12	0.055
**Alcohol Use (n, %)**	0, 0	4, 8	2, 8	3, 12	0.139
**Duration of DM (years)**	0	10 (5-15)	20 (7-20)	12 (7-19)	0.0001
**Duration of DN (years)**	0	0	2 (1-6)	4 (1.5-8)	0.178
**Hct (%)**	42(4)	40 (4)	40 (4)	40 (7)	0.247
HbA1c (mmol/mol)***	38 (36-40.7)	61 (50-74)	58 (50-84)	68 (52-98.5)	0.0001
**sTP (g/L)**	72 (3)	73 (4)	70 (5)	67 (7)	0.0001
**sAlb (g/L)**	46 (2)	45 (3)	43 (3)	40 (5)	0.0001
**sUrea (mmol/L)**	9 (7.5-11)	9.6 (8.2-12.8)	12.8 (8.9-16.2)	21.8 (12.5-31)	0.0001
**sCr (µmol/L)**	70 (58-80)	65 (56-77)	88 (71-119.5)	140 (94.5-204.5)	0.0001
**sCys C (mg/L)**	0.86 (0.79-0.95)	0.98 (0.86-1.16)	1.42 (1-1.84)	2.12 (1.47-3.43)	0.0001
**uTP (mg/24 h)**	192 (144-269)	123 (80-171)	185 (143-272)	1606 (853-2017)	0.0001
**uAlb (mg/24 h)**	4 (3-6)	5 (3-9)	77 (46-162)	1031 (530-1696)	0.0001
**uCr (mg/24 h)**	989 (710-1167)	1018 (844-1246)	893 (741-1302)	1047 (768-1351)	0.703
**CrCl (mL/min/1.73 m^2^)**	79 (58-107)	90 (72-105)	53 (43-92)	37 (18-75)	0.0001
**CKD-EPI-cr (mL/min/1.73 m^2^)**	93 (87-101)	97 (84-103)	71 (47-90)	44 (24-78)	0.0001
**MDRD (mL/min/1.73 m^2^)**	88 (78-99)	90 (78-101)	70 (47-85)	44 (24-71.6)	0.0001
**CG (mL/min/1.73 m^2^)**	84 (72-96)	90 (80-108)	64 (44-102)	48 (25-75)	0.0001
**CKD-EPI-cys (mL/min/1.73 m^2^)**	92 (82-101)	77 (61-89)	48 (33-76)	27 (14-27)	0.0001
**CKD-EPI-cr-cys (mL/min/1.73 m^2^)**	85 (93-104)	86 (70-100)	57 (38-79.5)	34 (20.5-56)	0.0001

* Data are reported as frequencies (%) for categorical variables and mean (standard deviation) or median (inter-quartile range) for continuous variables.

** BSA (body surface area) was calculated using the DuBois formula.

*** The relationship of HbA1c with the NGSP (%HbA1c) and the IFCC (mmol/mol) is: NGSP = [0.09148 * IFCC] + 2.152.

There was no significant difference between the groups in terms of gender (p= 0.064), age (p= 0.114), weight (p= 0.051), BSA (p= 0.25), duration of DN (for A2 versus A3 p=0.178), DBP (p= 0.621), and hct (p= 0.247). In all groups the percentages of non-smokers were between 84 ​​ and 100% and non-alcohol users were between 88 and 100%. The mean duration of diabetes in diabetic patients was 12.8 ± 8.9 years. There was no significant difference among the diabetic groups with respect to duration of DM. BMIs were significantly higher in the A1 (p= 0.0001), A2 (p= 0.0001), and A3 (p= 0.043) groups compared to the control group. Systolic blood pressures were significantly higher in the A1 (p= 0.003), A2 (p= 0.002), and A3 (p= 0.0001) groups compared to the control group. While HbA1c levels were significantly higher in the diabetic group (A1: p= 0.0001, A2: p= 0.0001 and A3: p= 0.0001) than the control group, the difference among the diabetic groups was not statistically significant. Serum total protein levels were lower in the A3 group than the control (p= 0.0001) and A1 (p= 0.01) groups. Serum albumin level was significantly lower in the A3 group than the control (p= 0.0001), A1 (p= 0.0001), and A2 (p= 0.009) groups. Serum creatinine levels were significantly higher in A3 group than all groups (Control: p= 0.0001, A1: p= 0.0001, A2: p= 0.006). Serum urea levels were higher in the A3 group compared to control (p= 0.0001) and A1 (p= 0.0001) groups. Serum cystatin C levels were higher in diabetic patients (A1: p= 0.024, A2: p= 0.0001, A3: p= 0.0001) than the controls, and in DN patients (A2: p= 0.028, A3: p= 0.0001) than the A1 group. Urine total protein and albumin levels were significantly higher in A3 group than the controls (uTP: p= 0.0001, uAlb: p= 0.0001). When compared all GFRs, only CKD-EPI-cys was significantly lower in A1 group than the controls (p= 0.021). All of the GFRs in A3 group were lower than control (CKD-EPI-cr, MDRD, CKD-EPI-cys, and CKD-EPI-cr-cys: p= 0.0001, CG: and CrCl: p= 0.001) and A1 (for all GFR p= 0.0001) groups. CKD-EPI-cr (p= 0.004), MDRD (p= 0.01), CG (p= 0.037), CKD-EPI-cys (p= 0.033), and CKD-EPI-cr-cys (p= 0.016) eGFRs in A2 group were significantly different from A1 group. The statistically significant differences between the subgroups in GFRs are shown in Table 2.

**Table 2:** Comparisons of GFRs between the subgroups

P Values	CrCl	CKD-EPI-cr	MDRD	CG	CKD-EPI-cys	CKD-EPI-cr-cys
**Control-A1**	1.000	1.000	1.000	1.000	0.021*	0.243
**Control-A2**	0.265	0.007*	0.012*	0.367	0.0001*	0.0001*
**Control-A3**	0.001*	0.0001*	0.0001*	0.001*	0.0001*	0.0001*
**A1-A2**	0.026*	0.004*	0.010*	0.037*	0.033*	0.016*
**A1-A3**	0.0001*	0.0001*	0.0001*	0.0001*	0.0001*	0.0001*
**A2-A3**	0.672	0.327	0.196	0.544	0.131	0.145

* p<0.05.

CKD patients were diagnosed when eGFR was less than 60 mL/min/1.73m^2^ 6. According to CKD-EPI-cr, MDRD, CG, CKD-EPI-cys, and CKD-EPI-cr-cys equations, the frequencies of the CKD patients were 31 (20.3%), 32 (21%), 38 (24.8%), 53 (34%) and 39 (25%) respectively. The mean eGFRs of CKD patients were 40.4 ± 15.3, 39.5 ± 14.7, 41.6 ± 13.8, 32.2 ± 15.2, 37.1 ± 15.2 according to CKD-EPI-cr, MDRD, CG, CKD-EPI-cys, and CKD-EPI-cr-cys formulas, respectively.

Correlations and p values between creatinine clearance (CrCl) and eGFRs in control and all diabetic subgroups are shown in Table 3.

**Table 3:** Correlations between CrCl and eGFRs

	CKD-EPI-cr		MDRD		CG		CKD-EPI-cys		CKD-EPI-cr-cys	
**CrCl**	**r**	**p**	**r**	**p**	**r**	**p**	**r**	**p**	**r**	**p**
**Control**	0.32	0.02*	0.44	0.001*	0.01	0.935	0.12	0.377	0.30	0.032*
**A1**	0.49	0.0001*	0.44	0.001*	0.48	0.0001*	0.40	0.004*	0.49	0.0001*
**A2**	0.84	0.0001*	0.83	0.0001*	0.84	0.0001*	0.70	0.0001*	0.82	0.0001*
**A3**	0.93	0.0001*	0.93	0.0001*	0.85	0.0001*	0.90	0.0001*	0.94	0.0001*

* p<0.05.

**Figure 1:**
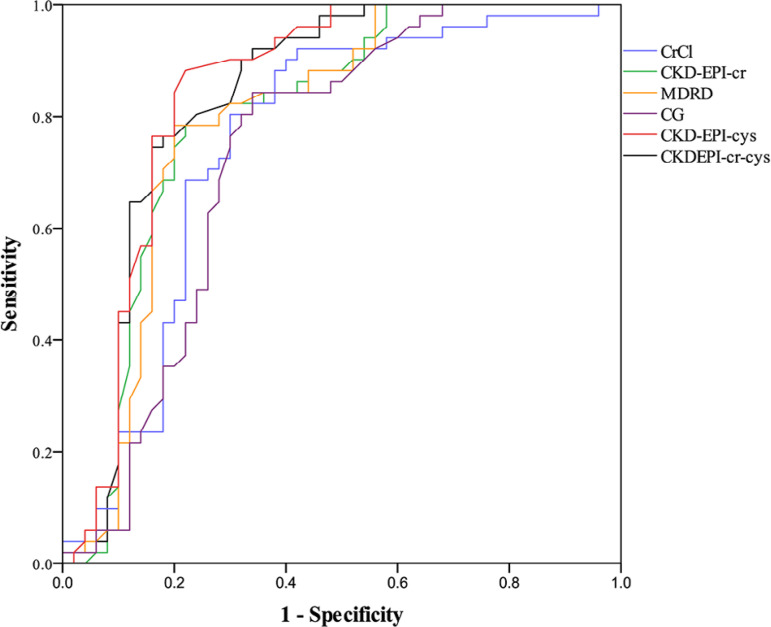
ROC curves for the prediction of diabetes nephropathy using CrCl and eGFRs.

CrCl, CKD-EPI-cr, MDRD, CG, CKD-EPI-cys, and CKD-EPI-cr-cys AUC values were calculated using ROC curve analysis between patients with DN (A2+A3) and normal to mildly increased albuminuria (A1): AUC_CrCl_= 0.755 (95%CI: 0.654-0.855, p= 0.0001), AUC_CKD-EPI-cr_= 0.799 (95%CI: 0.706-0.891, p= 0.0001), AUC_MDRD_= 0.795 (95%CI: 0.701-0.889, p= 0.0001), AUC_CG_= 0.734 (95%CI: 0.631-0.837, p= 0.0001), AUC_CKD-EPI-cys_= 0.847 (95%CI: 0.763-0.931, p= 0.0001), AUC_CKD-EPI-cr-cys_= 0.835 (95%CI: 0.749-0.921, p= 0.0001). The ROC curves are shown in Figure 1.

## Discussion

DN is one of the most important microvascular complications of diabetes mellitus, and causes high morbidity and mortality. Therefore, early detection of renal dysfunction is very important^16^. Serum and urine albumin levels can be used to evaluate renal functions. However, Epidemiology of Diabetes Interventions and Complications Study Group suggested that there are patients that progress to DN even without albuminuria^17^. The measured GFR (mGFR) is another good indicator for the evaluation of renal functions. However, more practical GFR formulas are widely used today, because the use of exogenous substances such as inulin or radioactive markers for measuring GFR are invasive and expensive methods that can lead to serious complications and high cost^2^. In our study, we have chosen the CKD-EPI-cr, MDRD, CG, CKD-EPI-cys, and CKD-EPI-cr-cys formulas, frequently encountered in the literature and recommended and used in practice^5^^,^ 6 ^,^ 7. Then, all eGFRs estimated using these formulas were compared to creatinine clearance instead of mGFR.

In our study, all eGFRs in patients with type 2 DN (A2, A3) were found lower than controls (see Table 1) in accordance with the literature^18^^,^ 19 ^,^ 20. The performances of CKD-EPI-cr and MDRD equations were similar to each other like Rognant et al. study^21^, although there are some studies suggesting that CKD EPI-cr performance is better than MDRD in diabetic patients^22^^,^ 23. The reason for these discrepancies may have been clinical features including age, BMI, and race^22^^,^ 23. While CrCl and CG eGFR values were lower in the A2 group than in control, these were not statistically significant, whereas the others were significant (see Table 2). Although patients were informed before the study, errors may have occurred while collecting 24-hour urine. Therefore, these errors may have negatively affected the results with CrCl^24^. Unlike other formulas, taking body weight in CG calculation may have caused eGFR values to be lower in controls compared to diabetic patients because of lower weight and BMI values in controls (see Table 1)^23^. We also found that all eGFRs except for CKD-EPI-cys in control group were lower than A1 group. Although all GFR formulas we used were indexed according to the BSA of 1.73 m^2^, these may have failed in reflecting real renal function in overweight and obese patients. It also should be noted that smaller individuals can have a lower normal GFR and larger individuals can have a higher normal GFR^25^^,^ 26. In addition, the patients in the early glomerular hyperfiltration stage of diabetic nephropathy may have caused high GFR values in A1 group. Hyperfiltration usually precedes changes in albuminuria in patients with newly diagnosed diabetes.^27^ Therefore, further formula improvements in discriminating between normal and hyperfiltration are needed.

Only CKD-EPI-cys levels in controls were significantly lower (p= 0.021) than A1 group. Many studies have suggested that cystatin C is comparable^28^ or superior^15^^,^ 29 to creatinine-based formulas in type 2 diabetic patients. Jeon et al.^30^ investigated MDRD, CKD-EPI-cr, and cystatin C levels in normoalbuminuric (n= 332), microalbuminuric (n= 83), and macroalbuminuric (n= 42) type 2 diabetic patients. Similar to our study, MDRD and CKD-EPI eGFRs were found significantly lower in the macroalbuminurics and microalbuminurics than in the normoalbuminurics (p<0.001). The cystatin C levels of serum and urine increased with increasing degree of albuminuria. Additionally, according to albuminuria, AUC value of cystatin C was 0.906. The authors briefly suggested that serum and urinary cystatin C levels are useful markers for renal dysfunction in normoalbuminuric type 2 diabetic patients. El-eshmawy et al.^15^ researched GFRs in 75 type 2 diabetic patients and 15 controls. Comparing macroalbuminurics (n= 25) to microalbuminurics (n= 25), they found that CKD-EPI-cys was significant (p>0.0001) while CKD-EPI was not. They also reported that AUC creatinine value (0.57) was lower than AUC cystatin C (0.79). Our findings were consistent with these studies and made us think that cystatin C could be more predictive in diagnosing early stages of renal dysfunction.

In the study of Kedam et al.,^18^ 239 type 2 diabetic patients (normoalbuminurics: 110, microalbuminurics: 81, macroalbuminurics: 48) were evaluated. The serum cystatin C levels were found negatively correlate with MDRD eGFR (r= -0.364, p<0.0001), and significantly higher in the macroalbuminurics than in the normoalbuminuric and microalbuminuric groups (both p<0.001), whereas they were not significantly different between the normoalbuminuric and microalbuminuric groups. The reason for these results may be that durations of DM in the normoalbuminuric and microalbuminuric groups were short and close to each other (5.0-7.5 years), as a long diabetes mellitus duration is one of the factors that increase the level of cystatin C leading renal damage^31^.

Bevc et al.^28^ used CrEDTA for gold standard GFR measurement in type 2 diabetic overweight patients (n= 113, BMI= 31.3±4.8kg/m^2^) and compared CrEDTA clearance to CG, MDRD, CKD-EPI-cr, and CKD-EPI-cys eGFRs. All eGFRs showed a significant correlation with CrEDTA clearance. In ROC analysis, AUC value was found highest in CKD-EPI-cys (AUC= 0.966). In our study, although CrCl was used instead of the gold standard method (mGFR) due to its cost and complications, eGFRs of all diabetic patients showed similar correlation with CrCl. CKD-EPI-cys had the highest AUC value (0.847) for prediction of DN. Unlike creatinine, this may explain that cystatin C is not affected by age, race, gender, muscle mass, and inflammation^32^. Unfortunately, cystatin C test prices are still higher than creatinine tests and this factor limits the use of cystatin C in routine laboratories.

While some researchers suggest using cystatin C for diabetic nephropathy, the others claim that it is not significant. For example, Iliadis et al.^33^ found that eGFR-cys is not better than eGFR-cr in 448 type 2 diabetic patients compared to mGFR (Cr-EDTA clearance). However, previous studies have shown that different reference methods used as mGFR can cause different results^34^. It should be also taken into account that creatinine clearance and various eGFR formulas determined and assessed with different gold standards can cause different eGFR results, so these formulas are not exactly comparable^35^.

The limitations in our study were as follows: first, we did not have a reliable gold standard for mGFR method because of its cost and complications. Moreover, sample sizes of the DN subgroups were too small. Additionally, our patient groups differed in terms of some medication and we did not have detailed information about whether these drugs affect renal function.

## Conclusion

CKD-EPI-cys eGFRs of all diabetics including A1 group were significantly different from controls, while CKD-EPI-cr, MDRD, CKD-EPI-cys, and CKD-EPI-cr-cys eGFRs in A2 group were significantly different from the A1 group. Our results showed that the CKD-EPI-cys eGFR had better predictive value than the others for DN and it can be useful in detecting the early stage of DN. More extensive cohort studies with more participants are needed for the widespread use of cystatin C in the evaluation of diabetic kidney function.
